# Oral florid papillomatosis: Topical treatment with 5% imiquimod in orabase

**DOI:** 10.1002/cre2.557

**Published:** 2022-04-13

**Authors:** Pedro Ruiz‐Huertas, Alicia Borrego‐Luque, Pilar Toledano‐Valero, Carolina Manzotti, Ángel Rollón‐Mayordomo

**Affiliations:** ^1^ Department of Oral and Maxillofacial Surgery Virgen Macarena University Hospital Seville Spain

**Keywords:** imiquimod, immunotherapy, oral florid papillomatosis, oral mucosa

## Abstract

**Background:**

Florid oral papillomatosis is characterized by its tendency to local recurrence that requires multiple treatments, leading to important functional sequelae.

**Methods:**

We present 74‐year‐old woman with oral florid papillomatosis (OFP) who refused a new surgical treatment, and was treated with imiquimod 5% in orabase on alternate days for 16 weeks. Treatment was complemented with application of hyaluronic acid gel.

**Results:**

There were no side effects to the treatment, nor signs of local recurrence, in the treated area at 2 years of follow‐up.

**Conclusions:**

After reviewing the literature and according to our knowledge, this is the first published case of oral florid papillomatosis treated topically with imiquimod 5% successfully. Topical treatment with imiquimod 5% in orabase may be a valid alternative for patients with recurrent OFP located in the anterior area of the oral cavity who refuse surgical treatment, although we must closely monitor the patient for the possibility of recurrence or malignant degeneration.

## 1 INTRODUCTION

Oral florid papillomatosis (OFP) consists of the formation of multiple verruciform and papillomatous growths that converge forming plaques and vegetations (Eversole, 2000). It occurs more frequently in men between 60 and 70 years. Tobacco is the most important etiological factor (Schwartz, 1995), followed by significant alcohol consumption, poor oral hygiene, trauma or chronic irritants, immunosuppression (Grillo et al., 2012), and human papillomavirus (HPV) whose most frequently detected serotypes are 6, 11, and 16 (Eversole, 2000). It is considered as a variant of low degree of malignancy of oral mucosal verrucous carcinoma and can sometimes develop areas of infiltrating squamous cell carcinoma (Wenzel et al., 2003), which require close monitoring and treatment aimed at definitive resolution (Yoshimura et al., 2001).

The treatment of OFP is eminently surgical by excision, electrocoagulation, and CO_2_ laser (López‐Jornet et al., 2019). Cryotherapy with liquid nitrogen and topical chemotherapeutic, cytostatic and immunomodulatory agents have also been used, including retinoids, topical salicylic acid, trichloroacetic acid, podophyllin, podophyllotoxin, 5‐fluoruracil, systemic cimetidine, inosine pranobex, alpha interferon, imodon, interferon resiquimod, dinitrochlorobenzene, diphencipron, intralesional bleomycin, cidofovir, and methotrexate  (Kwok et al., 2012).

The topical immunomodulatory drug imiquimod 5% cream has been used successfully for the treatment of exophytic lesions caused by HPV at the anogenital region (Gaspari, 2007; Wieland et al., 2006).

In the oral cavity, imiquimod has also been used for the treatment of different lesions (Allam et al., 2008; Gemigniani et al., 2015; Gencoglan et al., 2011; Martinez‐Lopez et al., 2017; Maschke et al., 2004; Méndez‐Flores et al., 2019; Mullins et al., 2016; Rinaggio et al., 2006; Spieth et al., 2006; Wenzel et al., 2003; Yasar et al., 2019).

Our objective is to assess as a pilot experience the use of topical Imiquimod 5% in orabase excipient in a patient with relapsing multifocal OFP located in the lower lip, oral vestibule, and tongue tip, after the failure of numerous surgical treatments.

## 2 MATERIALS AND METHODS

**Figure 1:**
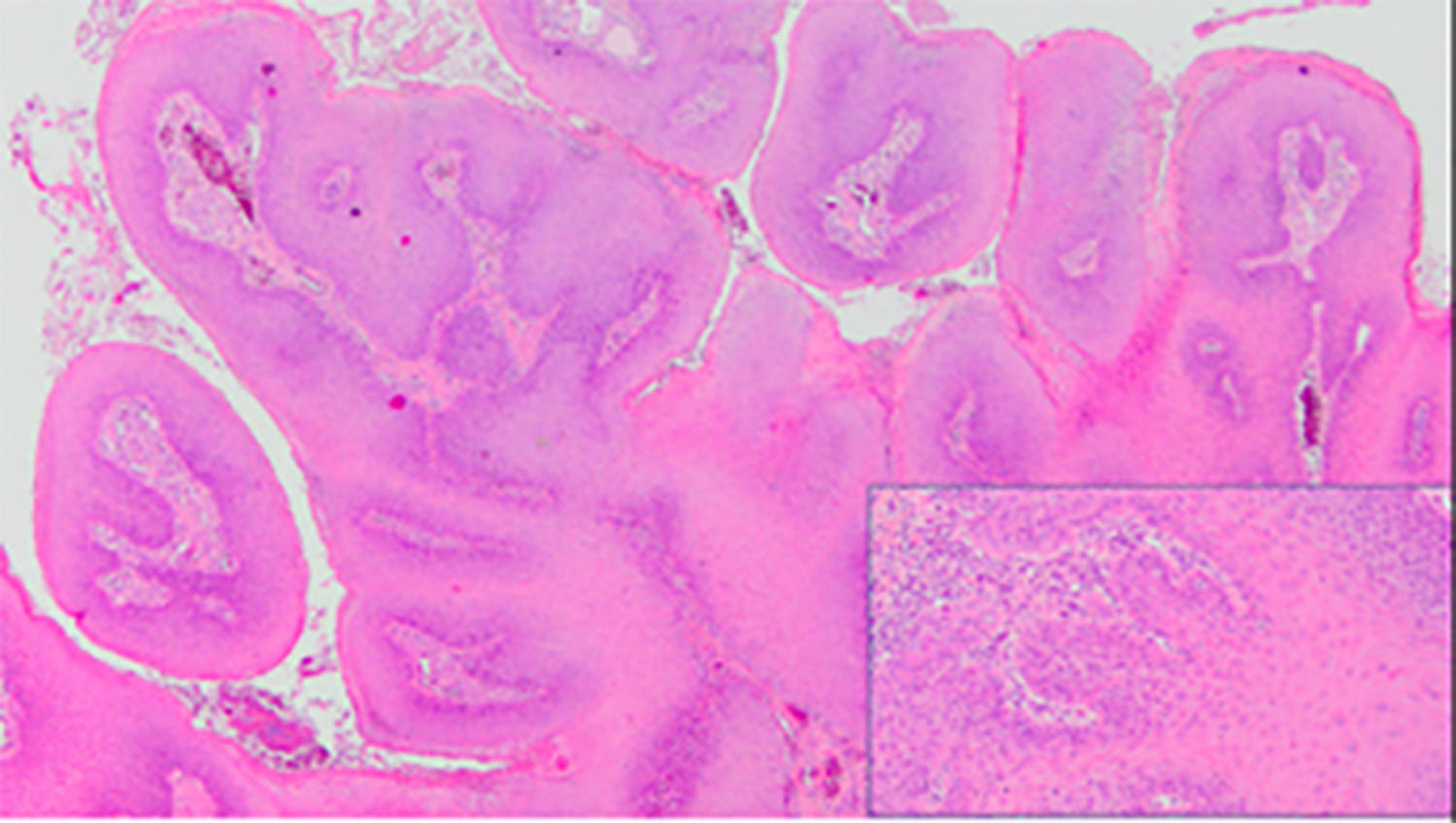
Histology of the last specimen removed shows papillary projections with fibrovascular cores, acanthosis, and hyperparakeratosis. Inset: Hyperplasia without epithelial dysplasia (hematoxylin‐eosin, ×20 and ×40)

**Figure 2:**
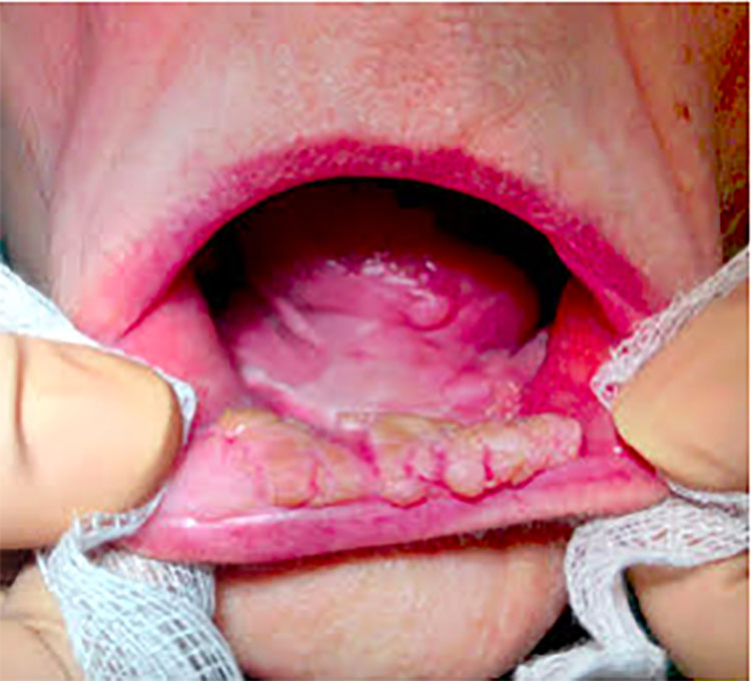
Lip and oral cavity with oral florid papillomatosis before treatment with imiquimod

A 74‐year‐old nonsmoking woman with multiple sclerosis was diagnosed in 2008 with OFP. Since then she has presented multiple recurrent lesions in the oral mucosa. The histological results of the lesions were keratosis with mild dysplasia on four occasions, papillary hyperplasia with mild atypia, proliferative verrucous leukoplakia, and verrucous carcinoma. The treatments performed were surgical excision or CO_2_ laser vaporization in dysplasias and surgical resection with oncological resection margins in the case of verrucous carcinoma. Histology of the last specimen removed showed papillary projections with fibrovascular cores, acanthosis, and hyperparakeratosis (Figure 1). The typing of the most common HPV species through a direct flow chip test that analyzes more frequent low‐risk and high‐risk viruses was negative. The patient was immunocompetent, did not perform immunosuppressive treatment for multiple sclerosis and her HIV serology was negative. In February 2017, the patient presented a new recurrence that affected the mucous edge of the lower lip, anterior oral vestibule, and tongue tip (Figure 2) whose biopsy was keratotic papilloma with mild atypia. The patient refused a new surgical treatment, which prompted us to seek another therapeutic alternative, so we proposed the compassionate use of 5% topical imiquimod, with the approval of the ethics committee of our hospital and the detailed informed consent signed by the patient.

**Figure 3:**
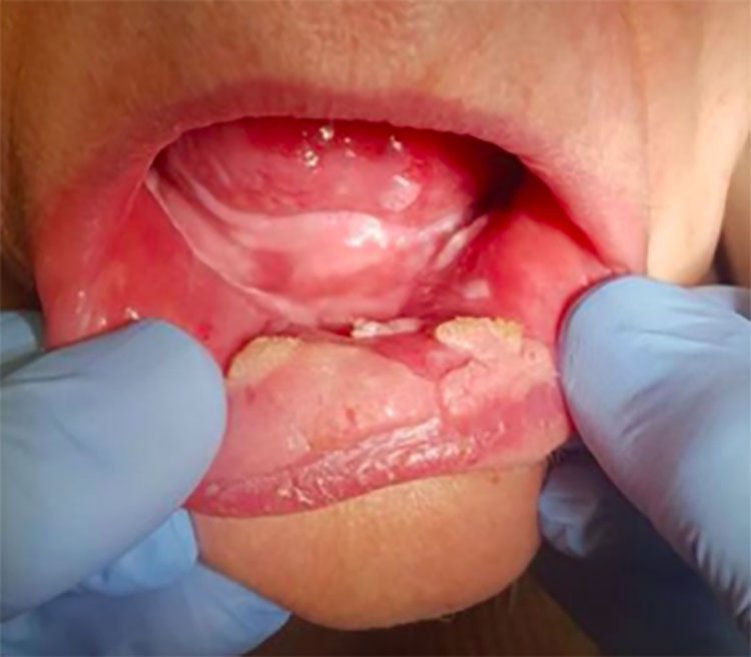
Lip and oral cavity 4 weeks after start of treatment

**Figure 4:**
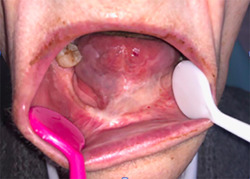
Lip and oral cavity 2 years after treatment

A formulation with 5% imiquimod was made in orabase. Our treatment regimen resembles the recommended guideline according to the leaflet for the treatment of anogenital verrucous lesions, the cream is administered three times per week (Monday–Wednesday–Friday) before going to sleep, with an applicator on the lesions to be treated. We recommend maintaining good oral hygiene, rinsing the mouth in the morning and applying gel with hyaluronic acid on the nontreatment days to minimize side effects on the mucosa adjacent to the lesions. A weekly check was carried out and good tolerance was observed (Figure 3), the treatment was maintained for 16 weeks (maximum period of use of treatment according to leaflet), confirming the complete disappearance of the lesions in this period of time. During the treatment, the patient had no side effects or complications. In subsequent reviews and after 2 years of treatment, the patient showed no signs of local recurrence of the disease in the treated area (Figure 4), she uses her dental prosthesis without discomfort and she can eat acid food, which used to cause severe irritation of her oral mucosa.

## 3 RESULTS

Topical treatment with imiquimod 5% in orabase may be a valid alternative for patients with recurrent OFP located in the anterior area of the oral cavity who refuse surgical treatment, although we must closely monitor the patient for the possibility of recurrence or malignant degeneration.

## 4 DISCUSSION

Imiquimod is an immunomodulator that lacks direct antiviral or antineoplastic effects. Its main mechanism of action is the promotion of the innate and adaptive immune response, by stimulating monocytes and macrophages to produce the release of a group of cytosines, such as interferon alfa, tumor necrosis factor, and interleukins 1, 6, and 8, and also gamma interferon and interleukin 12. All of the above leads to an increase in the immune response measured by Th‐1 lymphocytes, similar to delayed‐type hypersensitivity and induces proliferation and differentiation of B lymphocytes (Cianfriglia et al., 2006; Luo et al., 2007; Ostwald et al., 2003).

This drug in our country has been approved for the topical treatment of genital and external perianal warts, small superficial basal cell carcinomas and clinically typical actinic, nonhyperkeratotic, and nonhypertrophic keratoses of the face and scalp in immunocompetent adult patients. Before its use in oral mucosa, we conducted a literature review in Pubmed with the following keywords: “Oral florid papillomatosis”; “Imiquimod” and “Oral cancer”; “Imiquimod” and “Oral papillomatosis”; “Imiquimod” and “Leucoplakia”; “Imiquimod” and “Oral mucosa”; “Imiquimod” and “Lichen planus.” We found 11 articles that presented 17 patients with oral lesions treated with imiquimod.

The lesions described in these articles are focal epithelial hyperplasia in seven patients (Gemigniani et al., 2015; Maschke et al., 2004; Méndez‐Flores et al., 2019; Yasar et al., 2019), proliferative verrucous leukoplakia in one patient (Martinez‐Lopez et al., 2017), leukoerythroplasia and oral carcinoma in situ in one patient (Mullins et al., 2016), dysplastic oral leukoplakia in one patient (Allam et al., 2008), oral lichen planus in one patient (Gencoglan et al., 2011), and for papillomatous lesions and lichenoid cheilitis in a patient with a history of squamous cell carcinoma of the floor of the mouth (Wenzel et al., 2003). All these lesions that are sometimes not easy to differentiate histologically, have in common an instability or disease of the oral mucosa, which over time can develop squamous cell carcinomas. Imiquimod 5% has also been used for the treatment of the affected surgical margins of oral melanoma (Spieth et al., 2006) and bowenoid papulosis (Rinaggio et al., 2006).

In five patients, positivity was detected for the HPV (Allam et al., 2008; Gemigniani et al., 2015; Martinez‐Lopez et al., 2017; Rinaggio et al., 2006), in one patient the detection of the virus was negative (Méndez‐Flores et al., 2019) and in the rest of patients, there is no information on the possible viral etiology of the lesion. Another five patients had immunosuppression status: HIV‐positive (Méndez‐Flores et al., 2019; Rinaggio et al., 2006), leukocytopenia (Allam et al., 2008), and immunosuppressive treatment (Gemigniani et al., 2015).

The nondetection of HPV in our patient is consistent with other cases of POF where they also did not find the viral ethiology (Dias‐Polak et al., 2019).

In all the cases reviewed, topical imiquimod 5% cream without excipient was used. The most frequently used application is on alternate days and overnight; three times a week (Martinez‐Lopez et al., 2017; Spieth et al., 2006; Yasar et al., 2019), but also two nights a week in combination with 0.1% retinoic acid (Gemigniani et al., 2015), daily use (Mullins et al., 2016), two times a day (Gencoglan et al., 2011) or increasing exposure from 20 min to 2 h every 2 days has also been described (Allam et al., 2008).

The treatment weeks ranged between 2 and 16 weeks (Allam et al., 2008; Gemigniani et al., 2015; Gencoglan et al., 2011; Martinez‐Lopez et al., 2017; Maschke et al., 2004; Méndez‐Flores et al., 2019; Mullins et al., 2016; Rinaggio et al., 2006; Spieth et al., 2006; Wenzel et al., 2003; Yasar et al., 2019). Although in our patient the lesion disappeared at 14 weeks, we maintained the treatment for 16 weeks, as did Sirim et al. As it was well tolerated and it was the maximum recommended period in the leaflet.

The most frequent side effects described have been: inflammation (Martinez‐Lopez et al., 2017), erosion (Méndez‐Flores et al., 2019), ulceration (Méndez‐Flores et al., 2019), odynophagia (Mullins et al., 2016), enanthem (Allam et al., 2008), burning sensation (Spieth et al., 2006; Yasar et al., 2019), the persistence of lesions or appearance (Allam et al., 2008) of lip herpetic lip lesions (Yasar et al., 2019).

In 13 of the 17 published cases, the lesion disappeared without signs of local recurrence. A patient with lichen planus relapsed at 6 months (Gencoglan et al., 2011). Another patient with bowenoid papillomatosis discontinued the treatment at his own discretion to undergo cryotherapy (Rinaggio et al., 2006). A patient with a history of squamous cell carcinoma of the floor of mouth who was treated 4 years before with surgery, with papillomatous lesions and liquenoid cheilitis on the lip, suffered a degeneration to squamous cell carcinoma after 3 weeks of treatment, but the authors declare that the case was difficult to understand and could be related to the decrease in dermal dendritic cells (Wenzel et al., 2003).

In our case, and to increase the local action of the active substance and reduce possible adverse side effects in the adjacent oral mucosa observed in other studies, we reduced the concentration of imiquimod by 5% thanks to the excipient (orabase). This administration system is comfortable, it can improve the availability of the medication during the entire treatment process, prevent the loss of drug by mouth and protect the film from saliva and bioerosion produced by the tongue and teeth.

We supplement the treatment with hyaluronic acid gel on the nontreatment days to help regenerate the perilesional mucosa that may be affected by the use of the drug. All these measures have prevented the appearance of side effects and the need for a new surgical intervention while preserving the patient's quality of life. To our knowledge, this is the first case of OFP successfully treated with topical imiquimod 5%, with a 24‐month follow‐up period that is higher than that published by other authors whose range ranges from 3 to 18 months (Allam et al., 2008; Gemigniani et al., 2015; Gencoglan et al., 2011; Martinez‐Lopez et al., 2017; Maschke et al., 2004; Méndez‐Flores et al., 2019; Mullins et al., 2016; Rinaggio et al., 2006; Spieth et al., 2006; Wenzel et al., 2003; Yasar et al., 2019).

## AUTHOR CONTRIBUTIONS

**Pedro Ruiz‐Huertas**: Created the treatment hypothesis and designed the protocol. **Alicia Borrego‐Luque**: Responsible for clinical procedures and follow‐up of patients. **Ángel Rollón‐Mayordomo**: Analyzed the data and wrote the manuscript. **Pilar Toledano‐Valero** and **Carolina Manzotti**: Both the authors read and approved the final manuscript. All authors contributed toward data analysis, drafting and revising the paper and agree to be accountable for all aspects of the work.

## CONFLICTS OF INTEREST

The authors declare no conflicts of interest.

## Data Availability

The clinical relevance is based on the fact there are very few cases reported on oral pathology treated with imiquimod and this is the only case of oral florid papilomatosis treated successfully with a higher follow‐up than published cases.

## References

[cre2557-bib-0001] Allam, J. P. , Erdsach, T. , Wenghoefer, M. , Bieber, T. , Appel, T. R. , & Novak, N. (2008). Successful treatment of extensive human papillomavirus‐associated oral leucoplakia with imiquimod. British Journal of Dermatology, 158(3), 644–646. 18076703 10.1111/j.1365-2133.2007.08374.x

[cre2557-bib-0002] Cianfriglia, F. , Di Gregorio, D. A. , Cianfriglia, C. , Marandino, F. , Perrone Donnorso, R. , & Vocaturo, A. (2006). Incidence of human papillomavirus infection in oral leukoplakia. Indications for a viral aetiology. Journal of Experimental & Clinical Cancer Research, 25(1), 21–28. 16761614

[cre2557-bib-0003] Dias‐Polak, D. , Kra‐oz, Z. , Szwarcwort‐Cohen, M. , Barzilai, A. , & Bergman, R. (2019). A case of oral florid papillomatosis (verrucous carcinoma) with lack of evidence for human papillomavirus involvement. American Journal of Dermatopathology, 41(8), 617–619. 31335422 10.1097/DAD.0000000000001168

[cre2557-bib-0004] Eversole, L. R. (2000). Papillary lesions of the oral cavity: Relationship to human papillomaviruses. Journal of the California Dental Association, 28(12), 922–927. 11323946

[cre2557-bib-0005] Gaspari, A. A. (2007). Mechanism of action and other potential roles of an immune response modifier. Cutis, 79(4, Suppl), 36–45. 17508494

[cre2557-bib-0006] Gemigniani, F. , Hernández‐Losa, J. , Ferrer, B. , & García‐Patos, V. (2015). Focal epithelial hyperplasia by human papillomavirus (HPV)‐32 misdiagnosed as HPV‐16 and treated with combination of retinoids, imiquimod and quadrivalent HPV vaccine. Journal of Dermatology, 42(12), 1172–1175. 26047065 10.1111/1346-8138.12967

[cre2557-bib-0007] Gencoglan, G. , Nanir, S. , Sahin, O. , & Gunduz, K. (2011). Imiquimod 5% cream for isolated lichen planus of the lip. The Journal of Dermatological Treatment, 22(1), 55–59. 20524879 10.3109/09546630903456367

[cre2557-bib-0008] Grillo, E. , Miguel‐Morrondo, A. , Vano‐Galván, S. , & Jaén‐Olasolo, P. (2012). Oral florid papillomatosis. Revista Clínica Española, 212(11), e93. 23083695 10.1016/j.rce.2012.08.003

[cre2557-bib-0009] Kwok, C. S. , Gibbs, S. , Bennett, C. , Holland, R. , & Abbott, R. (2012). Topical treatments for cutaneous warts. Cochrane Database of Systematic Reviews, 2012(9), CD001781. 10.1002/14651858.CD001781.pub3 PMC8101088 22972052

[cre2557-bib-0010] López‐Jornet, P. , Camacho‐Alonso, F. , & Berdugo, L. (2019). Oral focal epithelial hyperplasia. New York State Dental Journal, 76(4), 54–55. 20863043

[cre2557-bib-0011] Luo, C. W. , Roan, C. H. , & Liu, C. J. (2007). Human papillomaviruses in oral squamous cell carcinoma and pre‐cancerous lesions detected by PCR‐based gene‐chip array. International Journal of Oral and Maxillofacial Surgery, 36(2), 153–158. 17110084 10.1016/j.ijom.2006.09.005

[cre2557-bib-0012] Martinez‐Lopez, A. , Blasco‐Morente, G. , Perez‐Lopez, I. , Naranjo‐Diaz, M. J. , Aneiros‐Fernandez, J. , Ruiz‐Villaverde, R. , & Tercedor‐Sanchez, J. (2017). Successful treatment of proliferative verrucous leukoplakia with 5% topical imiquimod. Dermatology and Therapy, 30(2)​. http://www.ncbi.nlm.nih.gov/pubmed/27647537 10.1111/dth.12413 27647537

[cre2557-bib-0013] Maschke, J. , Brauns, T. C. , & Goos, M. (2004). Imiquimod for the topical treatment of focal epithelial hyperplasia (Heck disease) in a child. Journal der Deutschen Dermatologischen Gesellschaft, 2(10), 848–850. 16281588 10.1046/j.1439-0353.2004.04751.x

[cre2557-bib-0014] Méndez‐Flores, S. , Esquivel‐Pedraza, L. , Hernández‐Salazar, A. , Charli‐Joseph, Y. , & Saeb‐Lima, M. (2019). Focal epithelial hyperplasia in adult patients with HIV infection: Clearance with topical imiquimod. Skinmed, 14(5), 395–397. 27871359

[cre2557-bib-0015] Mullins, R. , Ansell, M. , & Laverick, S. (2016). Treatment of oral dysplasia with 5% imiquimod cream: Short communication. British Journal of Oral Maxillofacial Surgeons, 54(9), 1028–1029. 27178528 10.1016/j.bjoms.2016.01.030

[cre2557-bib-0016] Ostwald, C. , Rutsatz, K. , Schweder, J. , Schmidt, W. , Gundlach, K. , & Barten, M. (2003). Human papillomavirus 6/11, 16 and 18 in oral carcinomas and benign oral lesions. Medical Microbiology and Immunology, 192(3), 145–148. 12920590 10.1007/s00430-002-0161-y

[cre2557-bib-0017] Rinaggio, J. , Glick, M. , & Lambert, W. C. (2006). Oral bowenoid papulosis in an HIV‐positive male. Oral Surgery, Oral Medicine, Oral Pathology, Oral Radiology, and Endodontology, 101(3), 328–332. 10.1016/j.tripleo.2005.02.066 16504866

[cre2557-bib-0018] Schwartz, R. A. (1995). Verrucous carcinoma of the skin and mucosa. Journal of the American Academy of Dermatology, 32(1), 1–21. 7822496 10.1016/0190-9622(95)90177-9

[cre2557-bib-0019] Spieth, K. , Kovács, A. , Wolter, M. , Bug, R. , Kaufmann, R. , & Gille, J. (2006). Topical imiquimod: Effectiveness in intraepithelial melanoma of oral mucosa. The Lancet Oncology (London), 7(12), 1036–1037. 10.1016/S1470-2045(06)70979-2 17138226

[cre2557-bib-0020] Wenzel, K. , Saka, B. , Zimmermann, R. , Gundlach, K. K. H. , Barten, M. , & Gross, G. (2003). Malignant conversion of florid oral and labial papillomatosis during topical immunotherapy with imiquimod. Medical Microbiology and Immunology, 192(3), 161–164. 12719972 10.1007/s00430-002-0172-8

[cre2557-bib-0021] Wieland, U. , Brockmeyer, N. H. , Weissenborn, S. J. , Hochdorfer, B. , Stücker, M. , Swoboda, J. , Altmeyer, P. , Pfister, H. , & Kreuter, A. (2006). Imiquimod treatment of anal intraepithelial neoplasia in HIV‐positive men. Archives of Dermatology, 142(11), 1438–1444. 17116834 10.1001/archderm.142.11.1438

[cre2557-bib-0022] Yasar, S. , Mansur, A. T. , Serdar, Z. A. , Goktay, F. , & Aslan, C. (2019). Treatment of focal epithelial hyperplasia with topical imiquimod: Report of three cases. Pediatric Dermatology, 26(4), 465–468. 10.1111/j.1525-1470.2009.00954.x 19689526

[cre2557-bib-0023] Yoshimura, Y. , Mishima, K. , Obara, S. , Nariai, Y. , Yoshimura, H. , & Mikami, T. (2001). Treatment modalities for oral verrucous carcinomas and their outcomes: Contribution of radiotherapy and chemotherapy. International Journal of Clinical Oncology, 6(4), 192–200. 11706557 10.1007/pl00012104

